# Breeding confusion: hybrid seeds and histories of agriculture

**DOI:** 10.1080/03066150.2023.2180357

**Published:** 2023-03-01

**Authors:** Helen Anne Curry

**Affiliations:** a School of History and Sociology, Georgia Institute of Technology, Atlanta, GA, USA; b Department of History and Philosophy of Science, University of Cambridge, Cambridge, UK

**Keywords:** hybrids, hybrid corn, green revolution, biotechnology, genetically modified crops, terminator seeds, plant breeding

## Abstract

Since the 1970s ‘hybrid seeds’ have been linked to many perceived perils of industrialized agriculture. This essay revisits the scholarship that helped produce a dominant critical assessment of hybrid seeds, situating its emergence in a series of events and interventions of the late twentieth century. It explores how the singular history of F1 hybrid corn inflected understandings of crop breeding and seed production in general, contributing to effective political mobilization against agroindustry as well as lasting confusion about the promises and pitfalls of distinct approaches to crop development and the nature of hybrid seeds.

## Introduction

Accounts of twentieth-century agricultural industrialization in the United States and beyond often point to the production and distribution of hybrid seeds as a pivotal development. There is good reason for this emphasis. The successful commercialization of F1 hybrid corn seeds in the United States contributed to an enduring shift in the role played by public scientists in crop development (Kloppenburg 2004; Fitzgerald 1990, 1993) and paved the way towards a transnational seed industry (Howard 2009). Nor is corn the only crop associated with profound, hybrid seed–induced agricultural transformations. The semidwarf wheat and rice varieties that underpinned the so-called Green Revolution of the 1960s and 70s may be more typically characterized as ‘high yielding varieties’ but have also often been described as ‘hybrids’ (for examples over several decades, see Chakravarti 1973, 319–320; Omo-Fadaka 1975, 29; Shiva 1993, 62, 119; Thompson 2007, 563).

Building on these associations, celebratory accounts of ‘modernization’ achieved through the spread of hybrid seeds have been accompanied since the 1970s by critiques of the same. Critical accounts have pinned many social and environmental problems arising from industrialized agriculture on hybrid seeds, describing how these seeds undermined farmers’ independence and livelihoods, accelerated the spread of monocultures, diminished crop genetic diversity, and encouraged consolidation of corporate control over the global food system (e.g. George 1979; Fowler and Mooney 1990; Shiva and Jalees 2006). This straightforward depiction of an agricultural evil unsurprisingly has its own critics, and not just among agroindustry allies. In an analysis of vague terminology muddying decision-making in agricultural development in the 1990s, the anthropologist Robert Tripp (1996, 51) observed that ‘no term is more misused … than “hybrid.”’ Tripp was concerned that this term, which had come to be associated almost exclusively with F1 hybrids (a concept I explain further below), was being used to describe the semidwarf wheat and rice varieties of the Green Revolution – even though these crops were *not* F1 hybrids.

Tripp, drawing on the influential analysis of the sociologist Jack Kloppenburg, located the roots of this confusion in a linguistic shift arising from the commercialization of hybrid corn seeds. In the 1930s and 40s, a general meaning of hybridization as ‘the cross breeding or sexual combination of two varieties’ had narrowed to indicate the crossing of two inbred lines to produce F1 seeds as in the case of hybrid corn (Tripp 1996, 51; see also Kloppenburg 2004, 68). In fact, the shifting meanings of ‘hybrid’ among plant experts has a longer history, with an even older definition referring primarily to crosses between species. For Victorian botanists and horticulturists, interspecific hybridization was a process that produced what some saw as freaks of nature, others as highly marketable innovations, and everyone as a subject ripe for discussion and debate (Coleman 2021; see also Kingsbury 2011). Nonetheless, the introduction of hybrid corn seed production marked a pivotal moment in the history of ‘hybrid’ as a descriptor for plants. Thereafter this label was linked not just to nature's monsters or breeders’ artifices (as in the Victorian era) but also a new class of seeds – and, crucially, to the emerging systems of industrial agricultural research and production from which F1 hybrid seeds arose.

The association of hybrid seeds with industrial agriculture offers a better explanation of the elision observed by Tripp than the imprecision of the hybrid label. As I discuss, in the case of Green Revolution–era semidwarf wheat and rice, their categorization as ‘hybrid’ was not typically intended to identify the biotechnical means used by breeders to produce these seeds. Hybrid was then, and remains now, less a description of a breeding technique and more a signifier of the political and technological modes of production with which seeds are associated. In this case, it signifies ‘modern’ seeds often associated (correctly or not) with monocultural production, widespread use of chemical inputs, fossil fuel dependency, and control by transnational agribusinesses. Its use as such extends from the seed activism of the 1970s that first linked the commercial production of hybrid corn seed with broader global transitions in agro-industrial development through to contemporary discussions that seek to understand the biology and politics of genetically modified (GM) crops and especially ‘terminator’ genes.

Below I examine in greater detail the history and politics behind the use of ‘hybrid’ as a description for crop seeds developed for industrial agricultural production in the twentieth and twenty-first centuries.^1^ I call particular attention to how the history of F1 hybrid corn has been used to explain or exemplify broader patterns of agricultural, technological, and economic change. In other words, I am interested in both the label ‘hybrid’ and the narratives that have historically accompanied this label. Existing scholarship makes clear that the labels appended to seeds matter, not only to farmers but also to scientists, politicians, activists, and consumers. Consider two commonly encountered descriptions of seeds: ‘modern’ and ‘genetically modified.’ Calling seeds ‘modern’ (or, relatedly, ‘improved’) has typically conferred political or professional legitimacy on those who provide these seeds – plant breeders, especially – and cast doubt over those who persist in cultivating or promoting ‘traditional’ types (e.g. Yapa 1993; Cullather 2004). Identifying seeds produced through various technical interventions as ‘genetically modified’ (GM) has helped activists in multiple geopolitical contexts slow the introduction or use of GM crops (e.g. Stone 2002; Luna 2020; Aga 2021). Of course, it’s not just the label that matters. The political effectiveness of a seed's label depends on the larger narrative associated with it and the extent to which various audiences find it persuasive (Flachs 2021). ‘Modern’ seeds are only potent objects when some combination of farmers, agricultural advisors, and policy makers have faith that professional plant breeders and the scientific institutions they work for create products that address agricultural needs. The mobilizing power of the label ‘genetically modified’ among consumers has depended on activists hewing closely to a single narrative about the social and ecological dangers of all such seeds. As I discuss here, the history of hybrid seeds is entangled with that of both ‘modern’ and GM seeds and reveals some of the routes through which these and related labels gained and lost social and political salience. It also reveals the work accomplished by scholars and activists critical of industrial agricultural transformations over several decades through their accounts of hybrid seeds.

Historians of agriculture, science, and development have called for ‘new narratives’ of the Green Revolution and agricultural ‘modernization’ more generally (Patel 2013; Kumar et al. 2017), including accounts that expand the temporal and geographic frames used to explore the past century’s profound agricultural changes (e.g. Harwood 2012; Schmalzer 2016; Rojas and Alejandra 2019; Kumar 2020; Soto Laveaga 2020). Within this literature, well-worn tales of North–South technology transfer – a narrative first promulgated as a celebratory assessment by scientists, governments, and philanthropies engaged in development work – have rightly come in for scrutiny (Soto Laveaga 2021; Nehring 2022; Picado 2020). Yet there is also room for historians to re-examine the more critical narratives of the Green Revolution that followed close on its heels and remain dominant influences in scholarly literature today. What assumptions did these accounts entrench, and with what consequences for historical understanding and present-day action? Critical accounts of the 1970s and 80s highlighted unacceptable inequities, injustices, and environmental harms arising from developments in crop science and embodied in seeds and seed production systems. As I argue here, these accounts also generated confusion – often, though not always, unwittingly – about the means by which different seeds are produced. This confusion has served the interests of those who have opposed the growth and consolidation of capitalist agroindustry and sought to erode the authority of its champions. It may also have foreclosed, or be foreclosing, alternate ways of understanding and using the knowledge and tools of plant breeding developed in the past century. As such these accounts deserve greater attention in the drive to craft new and more historically nuanced narratives of twentieth and twenty-first century agricultural change.

## Hybrid corn seed

As I indicated in the introduction, it is possible to start a history of hybrid plants and seeds and the controversies around them in the nineteenth century or even earlier; however, the debates relevant to my analysis have twentieth-century origins. I therefore begin with the event that Robert Tripp (1996) understood as pivotal in determining the meaning of ‘hybrid’ when used as a description for crop seeds or varieties: the development of F1 hybrid corn. Contrary to some accounts, this development did not single-handedly launch today’s transnational seed industry, as the production and sale of seeds for private profit has significantly older origins (Curry forthcoming). It did however create new dynamics in seed production and marketing. As many authors have described – including those whose work I summarize here – by eliminating on-farm reproduction of seeds (i.e. through farmer seed saving), the use of F1 hybrid corn seeds allowed producers to exert new control over the spread of branded ‘improved’ varieties of a key global crop. This in turn afforded those producers expanded economic opportunities, initiating profound changes in the seed industry.

The history of hybrid corn will be familiar to many readers, not least through the pathbreaking work of Deborah Fitzgerald (1990, 1993) and canonical treatment by Jack Kloppenburg (2004), but its basic details bear repeating. In the late-nineteenth and early-twentieth centuries, most corn varieties grown in the United States were what are called today open-pollinated: populations maintained without exercising direct control over cross-fertilization. Farmers and breeders typically used mass selection to make naturally variable populations more uniform. This method, compatible with open pollination, involved selecting seeds in bulk from desirable plants, for example those with a consistent color or an extra row of kernels, without knowledge of individual parentage. Sometimes breeders (including farmer-breeders) crossed two plants and tracked the inheritance of traits through subsequent generations; knowledge of individual plants’ pedigrees enabled them to select for plants with a combination of desirable characteristics and use these as the foundation of a new variety. Breeders could also control pollination to create inbred lines. By collecting and deliberately redistributing pollen, breeders ensured that corn plants deemed ‘superior’ fertilized themselves or siblings. Inbreeding made their desirable traits ever more prevalent in the population. However, it also decreased plants’ fitness over time and eventually diminished yields: a uniform field was not necessarily a profitable one.

Botanists and breeders were aware that in many species the cross fertilization of two genetically distinct parents produces more vigorous offspring, a phenomenon known as heterosis or hybrid vigor. In a series of experiments in the early 1900s, the US geneticist George Shull observed that crossing two inbred lines of corn typically produced an offspring more vigorous than either parent. Although a tantalizing approach to increasing farmers’ yields, the use of hybrid vigor in corn seed production appeared to be infeasible. The genetic heterogeneity of the vigorous ‘first filial’ or F1 generation meant it would not reproduce true to type from saved seeds, forcing farmers back to a seed seller each season. However, the effects of inbreeding depression on each parent line led to low yields of the seeds necessary to grow the F1 hybrid generation and so to offset the expense of producing hybrid seed, it would need to be sold at a high price. The increased yields would never repay farmers’ initial investment.

In the 1910s, scientists developed techniques that circumvented the problem of low seed production resulting from inbreeding depression, paving the way for a hybrid corn seed industry (Fitzgerald 1990; see also Crabb 1947; Crow 1998). A young breeder at the Connecticut Agricultural Experiment Station, Donald Jones, proposed that breeders start with four distinct inbred lines (A, B, C, and D) instead of two. Cross A with B and C with D to create two different first-generation lines, AB and CD, each of which displays hybrid vigor. Because these first-generation lines do not suffer from inbreeding depression to the same extent as the parent lines, they can produce enough seeds from which to create a second hybrid generation, ABCD. These seeds could then be offered for sale. The promise of this method of producing F1 hybrid seed corn was measured in yields per acre and, especially for seed companies, in profits. As I’ve noted, vigorous F1 plants – that is, hybrid corn – generated higher yields but obliged farmers to return to a seed seller each year for a fresh supply. This is because they didn't reproduce true to type. The genetic heterogeneity of the hybrids meant *their* offspring (the F2 generation) would be unlikely to show the same productivity. This characteristic made F1 hybrid varieties appealing to seed companies, as they would be rewarded for the extra labor and cost needed to generate the F1 seeds by the annual return of customers for a fresh supply.

The first business founded explicitly around the production and sale of hybrid varieties was the Hi-Bred Corn Company, launched in 1926 by the agricultural writer and publisher Henry A. Wallace and rebranded as Pioneer Hi-Bred Corn Company a decade later (Brown 1983). Thanks to vigorous promotion and favorable farm policies, hybrid corn varieties overtook their open-pollinated predecessors at a rapid pace in the years that followed, constituting nearly 100 percent of cultivation in the US Corn Belt by the end of the 1930s and nationwide by 1960 (Duvick 2001, 71). Corn yields per acre more than doubled in the same period, and about half of that increase is attributed to breeders crafting ever-more productive hybrid varieties (Duvick 1999, 20).

The creation of a commercially viable system for producing hybrid corn seeds was celebrated as a profound advance of modern scientific understanding and agricultural industry. In the assessment of the geneticist James Crow, figuring out how to harness heterosis to create higher-yielding crop varieties was ‘surely one of genetics’ greatest triumphs’ (Crow 1998, 923). In an early attempt to capture the history of hybrid corn, the journalist A. Richard Crabb similarly enthused, ‘The development of hybrid corn is truly one of the most important advances made in all the thousands of years since man first began cultivating special food-bearing plants’ (Crabb 1947, xv). This ‘triumph’ altered the relationship between US corn growers and their seeds and had profound long-term effects on seed development and marketing. As many have observed (e.g. Berlan and Lewontin 1986; Yapa 1993; Kloppenburg 2004), the development of F1 hybrid corn seed expanded the possibilities for seeds to be commodities bought and sold like other farm inputs (such as pesticides and many fertilizers) and implements (like tractors, balers, and combines). The existing market for seeds had been limited by farmers’ ability to reproduce seeds by growing those they purchased and saving some at harvest time to plant the following season. However, if the plants grown from saved seed did not breed true, as in the case of F1 hybrid corn, on-farm seed production would not be an option and the market for seeds would look more like that for pesticides or tractors, in which farmers depended on market availability. This proved a significant inducement to private seed companies.

Success commercializing F1 hybrid corn seeds quickly led professional breeders and seed companies to pursue the same strategy in other crops, at least where biology allowed (see examples in Coors and Pandey 1999). Biology did not always allow, however. Corn generates high seed yields: planting a single seed generates a significantly larger number of seeds in the subsequent generation. It is also an outcrossing plant whose cross-pollination is easily controlled, for example by removing the pollen-producing tassels from the top of the growing plant or placing bags over developing ears to prevent unwanted genetic mixing. Thanks to these qualities it was possible for companies to price F1 hybrid corn seeds such that they would profit and farmers would also make up the premium they paid for the seeds through a larger harvest. By comparison, despite scientists’ best efforts, it was and remains challenging to develop commercially viable F1 hybrid seed systems for self-pollinating crops, including wheat and rice. Although public and private hybrid wheat breeding programs have been active since the 1950s, F1 hybrids represented only about 0.2 percent of global wheat acreage in the 2010s (Gupta et al. 2019). Hybrid rice has been comparatively more successful. Chinese breeders developed the first commercially viable varieties in the 1970s; by the turn of the twenty-first century, hybrids accounted for more than 50 percent of Chinese rice production (Li and Yuan 2000) and, thanks in part to philanthro-capital, had begun to make inroads elsewhere (Taylor 2020). Still, its extent is hardly comparable to that of hybrid corn.

The history of hybrid rice is also incomparable to that of corn in terms of the political economy of its development. As the historian Sigrid Schmalzer (2016, 2017) has described, hybrid rice in China has its roots in Mao-era research that was significantly shaped by the Cultural Revolution of the 1960s and 70s. Although most recent accounts celebrate the scientist Yuan Longping as the father of hybrid rice (much as Shull and Jones are credited with inventing hybrid corn), earlier documents recount a different story in which hybrid rice emerged out of collective work across multiple sites and was made available through the mass mobilization of peasants in seed production. This narrative of F1 hybrid seeds created through Chinese (as opposed to imported) knowledge and peasant labor to sustain Chinese workers and preserve Chinese communism stands in stark contrast to the story of profoundly capitalist F1 hybrid corn seeds that served private interests (and eventually transnational corporations) rather than farmers’ needs.

## Hybrids of the green revolution

The Green Revolution wheat and rice varieties promulgated through international development programs in the 1960s and 70s were described as hybrids almost from the moment of their introduction. The pattern can be observed across assessments, whether positive or negative, and irrespective of authors’ disciplinary expertise. For example, in 1973, an economist extolling advances in agricultural science credited ‘new hybrid varieties’ with a three- to five-fold increase in per hectare yields in wheat, rice, and maize in the tropics and subtropics (Skorov 1973, 13). A geographer’s more nuanced textbook treatment of rural change declared, ‘At the heart of the Green Revolution are the new hybrid wheat and rice varieties’ (Grigg 1978, 61). Yet the semidwarf wheat and rice varieties were not produced and distributed as F1 hybrid seeds. So what did ‘hybrid’ mean when applied to these varieties? And what political work did this label do? As I discuss here, in the hands of champions of the Green Revolution, reference to the hybrid origins of varieties was often intended to highlight the technical ingenuity and labor of their creators. However, when used by those critical of the Green Revolution, it more often raised questions about the suitability of seeds for poor farmers and the ultimate beneficiaries of development aid.

A simple explanation of why the label hybrid was used in describing seeds associated with the Green Revolution is that it referred to the origins of new varieties in the act of hybridization. The wheat varieties typically attributed to US plant breeder Norman Borlaug and credited with generating impressive leaps in wheat production in India and Pakistan had emerged from hybridizations among varieties of varied origins. In 1917, Japanese breeders crossed a local short-straw wheat variety (Daruma) with a soft wheat variety (Fultz) imported from the United States. In 1924, the offspring of this hybridization (Fultz-Daruma) was crossed with yet another US import (Turkey Red); Japanese scientists under the direction of Gonjiro Inazuka continued pedigree breeding on the Fultz-Daruma-Turkey Red mix, making selections from successive generations up to the release of the ‘final selection’ as Norin 10 in 1935 (Lumpkin 2015, 14–15). In the 1940s, a US Department of Agriculture wheat breeder visiting Japan collected samples of Norin 10, and these were eventually shared with another wheat breeder, Orville Vogel, of Washington State University. In 1949, Vogel began hybridizing Norin 10 with varieties grown in the United States. He shared F1 seeds arising from two of these crosses with Norman Borlaug, who began hybridizing them with varieties grown in Mexico. The hybrid of Norin 10 with the US variety Brevor proved especially good in combination with several Mexican varieties, leading to promising F1 generations (e.g. Norin-Brevor-Yaktana 54) that were then subject to further crossing and pedigree selection by Borlaug and his colleagues over multiple generations before being released as new true breeding lines for farmers in Mexico in 1962 (Lumpkin 2015; see also Cullather 2010). The eventual uptake of Norin 10–derived varieties in Mexico and beyond was tremendous, with some varieties grown over astonishingly wide areas soon after their introduction (Byerlee and Moya 1993; for critical accounts of Borlaug’s wheat research see Perkins 1997; Soto Laveaga 2021; Baranski 2022).

The ‘miracle’ rice variety IR-8 similarly originated in bringing together distinct parent lines – in this case an Indonesian variety (Peta) and a dwarf variety from Taiwan (Dee-geo-woo-gen). In 1976 the rice breeder and IR-8 contributor Peter Jennings described the steps that followed on from this initial hybridization and others like it:

The resulting hybrid seed, which yields the F1 generation, is generally grown under protected conditions, but the F2 and later generations go to several outdoor nurseries and to greenhouses where the plants are exposed to controlled stress. In the F3 and later generations the performance of all selections is determined in the field under standard conditions … If a particular trait is being sought, such as resistance to a known virus, then a series of test screenings, involving several generations, is undertaken.

As Jennings emphasized, any of these procedures could ‘indicate the need for further genetic manipulation.’ A potential new variety would also have to be grown and evaluated in farmers’ fields under varying conditions. In other words, moving from genetic recombination through hybridization to a new variety was a multi-year process – and one that putatively resulted in a stable, uniform line whose seeds farmers could confidently save from season to season (Jennings 1976, 186; for critical accounts that touch on the development of IR-8 see Oasa 1981; Cullather 2004, 2010; Stone and Glover 2017).

One could reasonably argue that the description of Green Revolution wheat and rice as ‘hybrids’ captured the origin of these varieties in the hybridization of markedly distinct breeding lines. Drawing on this older and more general definition, ‘hybrid’ here might refer, for example, to Borlaug's recombination of tall Mexican wheat varieties with varieties from the United States – US varieties that in this case were also ‘hybrid’ lines produced a few years earlier by crossing American varieties with Norin 10 from Japan. Or it might refer to the origin of IR-8 in the combination of Peta and Dee-geo-woo-gen, even though this was followed by several generations of pedigree-informed selection to secure the stability of certain traits. In comparison to corn plants, which easily cross pollinate, rice and wheat are self-pollinating, making the act of hybridizing a painstaking technical feat and therefore subject to celebration on these grounds alone.^2^

However, as Robert Tripp (1996) observed, there was sometimes more to characterizing Green Revolution seeds as hybrid than simple adoption of a more encompassing definition. Thanks to its dominant association with F1 hybrids, this term ‘implie[d] serious restrictions on farmer seed saving’ (52). When used by some critics as a description for the Green Revolution wheat and rice varieties, ‘hybrid’ suggested that these seeds induced peasant farmers to abandon the practice of saving their own seeds – despite the fact that ‘[n]one of the rice or wheat varieties of the green revolution were [F1] hybrids … and evidence shows that farmers who plant M[odern] V[arieties] of these crops save the seed of a particular variety for many seasons’ (Tripp 1996, 52). In short, referring to Green Revolution seeds as hybrids was sometimes a rhetorical move rather than a technical description – one that positioned these ostensible products of international aid firmly in the realm of capitalist agroindustry.^3^ It was intended to raise questions about the true beneficiaries of agricultural development programs: Were these subsistence farmers eking out an existence on the margins? Or large landholders who could afford more commercial inputs, including seeds? Or the foreign investors and business owners who sought profits from farmers’ investments in ‘modern’ varieties?

Evidence that the description ‘hybrid’ for seeds could and did affect how they were perceived, promoted, and rejected – with lasting consequences for historical accounting as well as agriculture – comes from an area of Green Revolution–era seed production that *did* at times involve the production of F1 hybrids. In Mexico, the same joint Rockefeller Foundation–Mexican government program that employed the wheat breeder Norman Borlaug also sponsored a corn breeding program. Its corn breeders worked from the 1940s onward to develop higher-yielding varieties for Mexican farmers. Unlike Borlaug, who turned his efforts towards wealthier farmers with access to irrigation (Baranski 2022), those leading the corn program considered it essential to address the needs of smallholder farmers working rainfed lands (Matchett 2002). As a result, they deemed F1 hybrid seed production inappropriate: farmers needed to be able to harvest the next generation's seeds themselves (Matchett 2006). Still, the production of F1 hybrids had cache. As the historian Karin Matchett (2002) summarizes, for many involved in agricultural improvement in Mexico in the 1940s, ‘[F1] “hybrid corn” symbolized modern, scientific corn cultivation. Since the mandates of both agencies [the Mexican national agricultural ministry and the joint Rockefeller Foundation–Mexican program] were to modernize cultivation to increase corn production, breeders at both agencies felt pressure to discuss hybrids’ (145).

As a result of this pressure, the production of F1 hybrids continued to be an objective of leading corn breeders working for Mexico’s national agricultural research institutions. More to the point here, it also influenced the way seeds created through *other* breeding methods were presented to patrons and farmers. For example, corn breeders associated with the joint Rockefeller Foundation–Mexican government program sometimes described their early ‘synthetic’ varieties (created by mixing several inbred lines) and other open-pollinated products that originated in crossing distinct lines but nonetheless reproduced true to type as ‘hybrids,’ knowing this label would enhance their desirability (Matchett 2002, 147). An unintended consequence of this labelling was the later proliferation of accounts that accused the American-led corn breeding program of ignoring the needs of peasant farmers by focusing on F1 hybrid varieties whose seeds could not be saved (e.g. Hewitt de Alcántara 1976; see discussion in Matchett 2002, 10). As Matchett’s detailed reconstruction shows, this perspective – borne of interpreting ‘hybrid’ as ‘F1 hybrid’ and appending assumptions about seed saving to it – mischaracterized the program’s initial aspirations and technical efforts.

## Blighted hybrids

The saveability of seed came in for special scrutiny from the late 1960s onward. To understand why it is necessary to turn from questions of plant breeding method to those of intellectual property. Rather than recapitulate debates over whether and how plant breeders should be allowed to secure intellectual property protections in their creations – the subject of an exhaustive literature and familiar territory for many readers – I will zero in on the position that ‘hybrid seeds’ occupied in these debates.^4^ As I show, observers critical of the growing power of transnational enterprises over global agriculture and wary of states’ extension of formal intellectual property protections in plant varieties to serve these industries often drew on the history of F1 hybrid corn to develop arguments and mobilize action against global agribusiness and the enclosure of the seed commons. These accounts emphasized that the biology-enabled restrictions on seed saving present in F1 hybrids would be superseded by the document-enabled restrictions of plant patents and variety registrations – and that the same hazards that threated industrial corn would plague the expanded range of plants in which intellectual property could now be claimed.

In the 1970s, activists in the United States and Europe increasingly identified transnational capital as a chief source of problems in the global food system. This was linked to broader critiques of capitalism and its relationship to scientific and technological development. For example, industrial food and agriculture were frequently targeted by the Marxist activists associated with the organization Science for the People (Schmalzer, Chard and Botelho 2018, ch. 6). The March 1975 issue of *Science for the People* magazine, one of the first issues to prominently feature discussions of agroindustry, featured back-to-back accounts of the ‘concentration of power in the food business’ and its effects on eaters and workers in both wealthy and poor countries. US readers were warned that ‘increasing concentration in the food industry has a lot to do with the fact that working conditions, employment, and food quality are staying the same or going down, while food prices are going up’ (Union for Radical Political Economics 1975, 8). And although politicians and scientists often attributed hunger abroad to poor farming practices or adverse social and ecological conditions, readers were encouraged to factor in ‘that agricultural production is only indirectly related to feeding people, being instead an activity which is carried out primarily for profit’ (Dennis and Landsness 1975, 17). For political activists and many other observers, the dominance of big firms, and their concern only for the bottom line, increasingly explained global maldistribution of food, poor diets, and ecological degradation (see, e.g. Lappe and Collins 1977; George 1979).

A subset of activists of the 1970s interested in the intersections of science, agriculture, and development homed in on control over seed production as a key route through which the influence of transnational corporations in agriculture was deepening (Schurman and Munro 2006, 2010). Private industry had sought new means of exercising this control through intellectual property protections on crop varieties and were rewarded with the expansion of such protections in Europe (via the International Union for the Protection of New Varieties of Plants, UPOV) and the United States (via the Plant Variety Protection Act, PVPA) in the 1960s and early 70s. Intellectual property in seeds thus became an important – and enduring – site of contestation for many activists (Aoki 2008).

In his 1979 *Seeds of the Earth*, perhaps the most famous intervention into these debates, the Canadian activist Pat Mooney wove a complex narrative about the social and political changes that had led to ever-more-thorough dominance of industry in the supply of seeds to farmers and the social and environmental consequences of this new order (Mooney 1979). He dismissed the claimed benefits of the Green Revolution to peasant farmers and pointed instead to wealthy countries and, more specifically, transnational agribusinesses, as the real beneficiaries of agricultural industrialization in developing nations. Private seed companies had stepped in to produce and distribute new varieties. Those same companies had recently convinced many governments to expand the intellectual property protections to be given to plant varieties. Seed activists including Mooney (1979, 1983) linked the guarantees of expanded intellectual property systems to intensifying industry consolidation, in which large companies were buying out the small seed firms of an earlier era, to the loss of crop genetic diversity and ultimately to further erosion of poor farmers’ already limited autonomy through the control of seed production.

For those who sought to understand and illustrate where greater private ownership of crop varieties would eventually lead, US hybrid corn, and F1 hybrids more generally, provided the obvious example. The production of F1 hybrid seeds had appealed to private companies because it enabled them to exercise some control over the varieties they developed and sold even in the absence of formal intellectual property protections. Mooney claimed that this produced ‘the hybrid bias,’ a tendency for companies to emphasize crops amenable to F1 hybrid seed production. As a result, ‘what is fundamentally a marketing consideration eventually influences the range of food varieties offered to people’ (Mooney 1979, 84). F1 hybrid technology had also made seed companies more powerful. A couple decades after the introduction of hybrids, a few hybrid corn seed companies had grown from modest family businesses into large corporations with significant economic clout (Fernandez-Cornejo 2004). They proved potent politically, too, arguing successfully against the release of new corn varieties by publicly funded programs as unfair competition, thereby diminishing public investment in and oversight of crop development (Kloppenburg 2004). F1 hybrid corn seeds had paved the way for privatization.

In one of the most famous accounts of this progression, the sociologist Jack Kloppenburg (2004; first published in 1988) outlined two transformative outcomes of the invention of hybrid corn. When seed production by private companies displaced on-farm production, the seed itself became a commodity. When these same firms shunted public programs to a supportive rather than competitive role, they established control over the form this commodity could take. The upshot, in Kloppenburg’s estimation, was a system that afforded less autonomy to farmers, diminished government research, transformed public investments in agriculture into subsidies for private companies, and reinforced industrialized agriculture through the dissemination of varieties particularly suited to this style of farming (Kloppenburg 2004, 128; see similar analysis in Berlan and Lewontin 1986).

Critics like Mooney and Kloppenburg were also able to rely on the example of US hybrid corn to illustrate the hazards of an agricultural system possessing these features. In the 1960s, bountiful corn harvests were not only central to the agricultural economy and domestic food prices but also a means by which politicians exercised ‘food power’ over foreign leaders (MacDonald 2016). The flow of cheap grain actualized US geopolitical strength. This vaunted position intensified the real and perceived effects of the Southern Corn Leaf Blight epidemic, a virulent fungal infection that swept US corn fields in 1970. The blight dramatically reduced harvests that year, with economic losses estimated at more than one billion dollars (the equivalent of 6.25 billion in 2022), and generated fears of feed shortages and escalating food prices (Ullstrup 1972). The blight was ultimately linked to the widespread adoption of hybrid lines that possessed identical cytoplasmic genes for male sterility. This was a quality that made hybrids cheaper for seed companies to produce but held no other advantages. In 1970, some 85 percent of hybrid corn grown in the United States shared the genetic material – originally sourced from a lone corn plant discovered in Texas to possess male sterility – that made them highly blight-susceptible (Ullstrup 1972, 39).

That biological explanation of the blight quickly gave rise to more political ones. ‘The proliferation of brand name maize varieties had disguised the fundamental genetic uniformity of corporate seed corn development,’ Mooney (1979, 11) declared, a circumstance that had led farmers unwittingly to plant all the same lines at significant peril to their livelihoods. The blight and its consequences substantiated his claim that ‘when these giant corporations direct their research dollars to hybrid development’ the result was ‘increasing crop uniformity and genetic vulnerability’ (70). And where some observers (mostly prominently, President Richard Nixon) were quick to praise the fast response of the nation’s public agricultural research system to the blight, Kloppenburg (2004, 122) later countered it was this public system’s ‘subordination to private enterprise,’ a condition typified in the production of F1 hybrid seed, that had endangered the nation’s corn harvest in the first place.

## Hybrid to GM

Activist agitation against the expansion of intellectual property rights in plant varieties was caught up in a distinct but related battle also just emerging in the 1970s: debates over the use and governance of new genetic technologies (Wright 1994; Purdue 2000; Schurman and Munro 2010; Stone 2010). The creation of recombinant DNA molecules generated by splicing the DNA of two distinct organisms and the development of ‘transgenic’ microorganisms that could possess and express genetic traits transferred through this technique indicated the possibility that transgenic crop varieties would soon be made as well. And while some observers worried about the chilling and distorting effects that the rapidly burgeoning private biotechnology industries would have on university biological research (Hughes 2001), seed activists focused on how the convergence of biotechnological prowess and strong intellectual property rights would shape the future of food (Schurman and Munro 2010; Purdue 2000). As I discuss, activists used the example of hybrid corn to illustrate the threats to farmer autonomy, narrowing control over crop development, and food system instability that might follow from widespread use of transgenic biotechnologies in crop development. They also coined an evocative and enduring label for a subset of biotechnologies that involved genetic barriers to seed saving (‘terminator’ seeds), again explaining the function of such seeds through a comparison to F1 hybrid corn. Using hybrid seeds to mobilize concern about ‘genetically modified’ and ‘terminator’ seeds proved effective – so effective that today many people fail to see meaningful differences between these categories of seed and stand steadfastly in opposition to them all.

In understanding these transitions, it is important to recall that Jack Kloppenburg’s trenchant analysis deployed the history of hybrid corn not to speak to the perils of corporate control of food and agriculture in general but to deliver a specific message about biotechnology. Writing in the 1980s as the new biotechnologies of recombinant DNA and other molecular interventions approached commercial viability, he urged readers against ‘ceding to capital the exclusive power to determine how biotechnology is developed and deployed’ (Kloppenburg 2004, 279). He predicted that biotechnologies used in breeding, bolstered by robust intellectual property protections, would ‘parallel the introduction of hybrid corn’ in further commodifying the seed, giving industrial actors the power to determine the shape of the crop as well as its production system, and committing farmers to a relentless technological treadmill (282–283). Kloppenburg’s analysis, like other critical takes on the use of transgenic biotechnologies in plant breeding, countered the claims of biotech companies like Monsanto, which initially maintained that its development of transgenic crops represented a clean, green break from their past – and certainly not business as usual (Glover 2010). It also intersected with a continued, powerful critique of the ‘modern’ and ‘improved’ seeds of the Green Revolution that focused on the extent to which new seeds had exacerbated rather than ameliorated the problems of poor farmers in Mexico, India, and other countries that had adopted them (e.g. Cleaver 1972; Griffin 1974; Hewitt de Alcántara 1976; Jennings 1988; Shiva 1993).

The comparison between F1 hybrid corn and transgenic varieties was made even more compelling a decade later, following the successful patenting of a ‘genetic use restriction technology’ by a US agribusiness, Delta & Pine Land Company, and the USDA in 1998 (Lombardo 2014). Seeds that deploy genetic use restriction technology – none of which have ever been commercially produced anywhere in the world thanks to vigorous international protest and moratoriums against them – are better known to most people as ‘terminator seeds’ or sometimes ‘suicide seeds.’ Genetic use restriction technologies (GURTs), of which there are multiple forms, involve transgenic manipulations that would enable the creator of the seed to restrict farmer seed saving. This could occur through the engineering of a biological mechanism that disrupts fertility or inhibits seed development (hence, terminator or suicide seeds) or the engineering of a desired trait that can be (de)activated through an external treatment (Lombardo 2014). In either case, a farmer would be unable to plant a second-generation crop from the initial purchase of seeds.

When Pat Mooney and colleagues at the Rural Advancement Fund International (RAFI, today ETC Group) first presented a case against ‘terminator technology,’ they drew a straight line from F1 hybrid corn seed to the looming GURT seeds. They reminded readers how, in 1908, the plant breeder and early conceptualizer of hybrid corn ‘George Shull came up with … a biological weapon to keep farmers from saving and developing their own seeds.’ In 1998, they warned, ‘[e]xactly 90 years after Shull's revelation, one of the biggest and most powerful of those companies, Monsanto, is fighting for control of the most important seed monopoly technology since the hybrid’ (Steinbrecher and Mooney 1998, 276). This assessment referred to the immediate interest shown by Monsanto in acquiring Delta & Pine Land Company and its promising patent. Monsanto's move was a vivid reminder that terminator seeds would combine forms of intellectual property: biological protections akin to those achieved in F1 hybrids as well as restrictions on who could develop GURT seeds through the enforcement of a patent.^5^ It also called attention to the fundamental prioritization of profit on the part of a company that had tried hard to sell itself and its agricultural biotechnologies as ‘pro-poor’ (Glover 2010).

The confluence of critical commentary linking F1 hybrid corn and later biotechnological developments, some scholarly and some not, ultimately contributed to an enduring conflation between F1 hybrid seeds and genetically modified (GM) varieties. Today it is common to encounter these diverse products of professional plant breeding lumped together and set in contrast with open-pollinated, locally adapted varieties known variously as heirloom, traditional, heritage, or landrace crops. For example, the US heirloom vegetable conservation organization Seed Savers Exchange characterizes its offerings as ‘Heirloom, Untreated, Non-Hybrid, Non-GMO Seeds’ (Seed Savers Exchange 2021).^6^ This aggregation of seeds produced through varied techniques of plant breeding into a single category of undesirable industrial seeds frustrates many people. A quick internet search reveals dozens of pages that explain the differences between hybrids and genetically modified seeds (e.g. Vinje n.d.; Mattern n.d.; Brogren n.d.). Often these authors seem convinced that basic education in the biotechnologies of seed production will dispel misunderstandings that they assume contribute to consumers’ rejection of crops produced through ‘modern’ breeding methods. This conviction misses the decades of work, unlikely to be easily undone, that has gone into developing a singular account of those breeding methods and the people and industries that benefit from them.

## Conclusion

Like confusion over the ‘hybrids’ of the Green Revolution, today's muddling of F1 hybrids with the products of more recent biotechnologies is a product of scholarly research and activist commentaries that deploy the history of F1 hybrid corn as a cautionary lesson against the expansion of intellectual property rights in seeds. These interventions – indebted to Marxist critiques of science under capitalism, entangled in international seed activism, rendered unusually compelling by the 1970 corn blight epidemic, and caught up in concern over biotechnologies – made hybrid corn seeds an incomparable example of the ills attendant on private industry's control of seed production. They undoubtedly contributed to the use of ‘hybrid’ as a generic negative description of the seeds associated with the Green Revolution. They also forged an enduring connection between hybrid seeds and those that would one day be produced through transgenic manipulation. These connections and elisions helped to mobilize action against the introduction of new seed technologies over several decades (Stone 2002). They are a crucial part of the story of the long Green Revolution (Patel 2013) and resistance to its many instantiations.

As I have shown, it was not a linguistic shift in response to the introduction of commercial F1 hybrids that created terminological confusion about hybrids, as Robert Tripp assumed. Contestation in the 1970s and later over the control of seeds – and with it the shape of global food system – generated the conditions in which ‘hybrid’ would be less a technical description of a seed's origin and more a potent shorthand for the style of agricultural production associated with it. For F1 hybrids, Green Revolution varieties, *and* GM crops, this was input-intensive and capital-dependent monocrop production that placed industry interests first.

This is an ironic outcome, to say the least. The foundation of arguments like Kloppenburg's, which equated the control enabled by of F1 hybrid production with that afforded by plant variety protection or patents, depended on an appreciation of the biological and technical specifics of F1 hybrid seed production. But the shorthand that runs roughshod over these specifics clearly endures – and is unlikely to be upended by more rigorous education in the breeding methods that produced them. By the end of the twentieth century, F1 hybrid corn seeds had been thoroughly entrenched as *the* historic lens through which to understand industrialized crops, regulatory regimes, and novel plant breeding technologies. They influenced – and continue to influence – assessments of the (not F1) hybrids of the Green Revolution, of varieties protected by patents and other intellectual property regulations, and ultimately of transgenic varieties including those that incorporate genetic restriction use technologies.

However useful and productive the comparisons have been over the past fifty years, it is surely worth pausing to reflect on whether we critics of agricultural modernization want to lean so heavily on hybrid corn as the go-to villain (e.g. Pollan 2006; Ramey 2010; Hubert 2017). First, as the examples of semidwarf wheat and rice illustrate, impressively genetically uniform monocultures can arise even without the close control over seed enabled by F1 hybrid technology or formal intellectual property protection. The case of F1 hybrid rice in China also raises questions about whether the spread of F1 hybrids can be ascribed solely to the engagement of capitalist enterprise in seed production (Schmalzer 2016, 2017). These examples serve as reminders that monocultures need to be explored and explained through their own distinctive biological, geographical, political, and cultural histories (as many scholars have argued, e.g. Uekötter 2014; McCook 2019; Robins 2021). Second, eliding the differences among different breeding methods may be an effective way of galvanizing concern about new biotechnology, for example the introduction of CRISPR-Cas9 and other methods of gene editing. But could it also foreclose possibilities for new alignments in the agricultural sector between biotechnologists and proponents of alternative agricultural futures (Clapp and Ruder 2020; Montenegro de Wit 2022). Finally, and perhaps most worrying, generalized negative perceptions of professional breeding and its products (as reported, e.g. in Barber 2015) may even undermine support for public sector breeding – a once robust but now deeply eroded enterprise that is also the only alternative we have to the privatization of seed development and production (Shelton and Tracy 2017).

## Footnotes

1 I do not attempt to link this with the literature on hybridity as it relates to human social, cultural, and genetic mixing, e.g., Brah and Coombes (2000), Hutnyk (2005), or Burke (2009).

2 For an account of how interest in the technical achievement of creating interspecific rice hybrids shaped rice research in Africa, see Maat (forthcoming).

3 There are good reasons to see these endeavors as historically aligned; see Jonathan Harwood (2019). My analysis is not intended to call this association into question.

4 Useful entry points into the history of intellectual property claims in plants include Bugos and Kevles (1992), Fowler (1994), Kloppenburg (2004), and Aoki (2008).

5 This layering of intellectual property controls was not new but already applied to any F1 hybrid varieties registered patented or registered under plant variety protection regimes.

6 An example of an extended account that relies on this contrast (i.e., hybrids and genetically modified crops *versus* heirlooms) is Ray (2012).
